# Qualitative exploration of barriers and enablers to migrant access to water safety programmes in Australia

**DOI:** 10.1136/bmjopen-2025-107233

**Published:** 2025-11-04

**Authors:** Jagnoor Jagnoor, Justin Scarr, Medhavi Gupta

**Affiliations:** 1 Injury Division, The George Institute for Global Health, Sydney, New South Wales, Australia; 2 Royal Life Saving Society Australia, Broadway, New South Wales, Australia; 3 Injury Division, The George Institute for Global Health India, New Delhi, India

**Keywords:** PUBLIC HEALTH, Awareness, QUALITATIVE RESEARCH, Wounds and Injuries

## Abstract

**Abstract:**

**Background:**

Migrants in Australia are a vulnerable group to drowning, yet their participation in water safety programs remains limited. Previous research has focused on migrants already engaged in water-related activities, overlooking perceptions from those facing broader access challenges. Additionally, migrants have often been treated as a homogeneous group, neglecting intersectional factors such as age, gender, ethnicity and income level that influence access to programs.

**Methods:**

This phenomenological qualitative study addresses these gaps by employing a novel approach to recruitment: conducting in-depth interviews with purposively selected recent migrants in a non-traditional setting—hairdressing salons—to capture diverse perspectives. A variety of ethnicities, ages and genders were recruited. Thematic analysis with an inductive approach was used to analyse the data.

**Results:**

Findings showed that most migrants were unaware of water safety programmes. While acknowledging the importance of water safety, they prioritised other pressures, such as income generation. However, affordable and culturally tailored training programmes for adults, delivered in culturally safe environments, may enable participation. Social connections, especially among students, could also be leveraged. Although parents rarely participated, they prioritised enrolling their children in swim training. Ethnicity-specific adaptations, such as native-language trainers, were considered desirable. Informants suggested disseminating water safety information to migrants before arrival in Australia or through community magazines and universities.

**Conclusions:**

This study highlights the importance of intersectional, community-driven designing of water safety programmes and demonstrates the effectiveness of innovative recruitment methods in reaching underrepresented migrant populations. These findings provide actionable insights for developing inclusive and accessible drowning prevention strategies.

**STRENGTHS AND LIMITATIONS OF THIS STUDY**

- This study employs a novel recruitment approach by seeking and interviewing informants in a non-water related setting, providing a wider migrant community perspective.
- Purposive selection was used to ensure that a variety of ethnicities, ages and income levels were recruited to identify diverse enablers and barriers.
- Thematic analysis with an inductive approach was used to analyse the data.
- Our findings were limited to broader experiences of migrants, but could be deepened by a larger sample size allowing the analysis of sub-groups along intersecting demographic axes.

## Introduction

 Water safety is a critical public health issue, as drowning imposes a significant economic burden and has profound consequences on individuals, communities and broader society.^1^ While high-income countries such as Australia have seen overall reductions in drowning rates, ethnic minorities and migrant populations remain disproportionately affected.^1^ This disparity highlights the need for targeted interventions to address the unique risks faced by migrants.

Australia’s diverse population, with 29% of residents born overseas and 20% having at least one parent born overseas, underscores the importance of inclusive water safety strategies.^2^ A report spanning 2005 to 2015 found that migrants accounted for 27.3% of drowning deaths (1.15 per 100 000 population), with long-term residents comprising 40% of these fatalities.^3^

Beyond Australia, studies from the Netherlands and Sweden reported an increasing proportion of drowning incidents among new migrant populations.^4 5^ In Sweden, migration has been described as reversing long-term downward trends in drowning mortality. These disparities emphasise the need for targeted interventions to address water safety among migrant populations, who face unique risks and vulnerabilities.

Migrants’ vulnerability to drowning is often linked to unfamiliarity with new geographical, social and cultural environments, coupled with limited access to water safety programmes. For instance, 41% of overseas-born residents in Australia have been classified as poor swimmers, with many reporting low levels of interest or prioritisation of water safety training.^6^ These findings suggest that cultural, social and systemic barriers hinder migrants' ability to access life-saving water safety programmes.

Although previous research has identified some barriers and enablers to migrants’ participation in water safety initiatives, these studies largely focus on individuals already engaged in such programmes or present in aquatic settings.^7^ This presents a biased perspective, overlooking migrants who are unaware of or unable to access these programmes. To develop inclusive and effective interventions, it is essential to understand the broader migrant population’s perspectives, particularly those who delay or avoid participation in water safety programmes.

The study aims to bridge this gap by exploring the barriers and enablers to water safety programme participation among migrants in Australia. By adopting community-based recruitment methods and an intersectional lens, this research seeks to uncover how factors such as ethnicity, gender, income and occupation influence access to water safety programmes. Ultimately, these insights will inform the design and implementation of more inclusive, culturally sensitive and accessible interventions tailored to migrant populations.

## Aims

This research aims to identify barriers and enablers to migrants accessing water safety programmes in Australia. We aim to capture a broad range of perspectives, particularly from those who have limited access to water and water safety programmes. In addition, we aim to identify any intersectional differences in barriers and enablers, including factors such as age, gender, occupation, income level and ethnicity. Identifying barriers or facilitators will help inform the development and implementation of future water safety plans better suited for migrant populations. The protocol for this study can be found in online supplemental file 1.

## Methodology

### Setting

In order to identify barriers and enablers of a wider range of migrants, including those who rarely access water settings, informants were approached at a number of hairdresser salons across metropolitan Sydney, Australia (see online supplemental file 2 for list of suburbs in which local salons were approached). New South Wales, the state within which Sydney is located, accepts new migrants in patterns largely representative of the rest of Australia.^8^ This novel approach to recruitment outside traditional healthcare settings was shown to be successful in a study by Victor *et al* 2018 in an ethnic minority group.^9^

### Study Design

The study applied a phenomenological approach to capture the informant’s lived experience. We conducted structured in-depth interviews (IDIs) (see online supplemental file 3 for interview script) to understand migrant population perspectives on knowledge and access to water safety programmes and facilities. The COREQ guidelines were used to guide study methodology (online supplemental file 4).

### Informants and sample size

Informants were recently migrated individuals who had settled in Australia for more than 6 months, but less than 7 years. To be eligible for the study, potential informants were over the age of 18 and born in one of the following countries: South Korea, Nepal, China, India or another migrant country if they spoke the following languages: English, Simplified Chinese, Traditional Chinese, Hindi, Korean, Arabic or Nepali. These groups have faced higher rates of drowning within the migrant community.^3^

Hairdressers at participating salons were trained by the research team to introduce the aims of the study to their clients. If a potential informant was interested, they were screened against eligibility criteria by a research assistant, and, if eligible, they provided informed consent.

Informants were offered a discounted haircut for their participation. For the purposes of this study, preventions were defined as swimming lessons, water safety training and cardiopulmonary resuscitation (CPR) training, as well as exposure to formal (university, school, sport and recreational clubs, societies, Royal Life Saving Society Australia) educational training or material on water safety.

In accordance with best practice guidelines for qualitative research, sample size was not determined a priori but was determined based on quota, where at least two informants of each ethnicity were acquired, and at least four informants were recruited for each age, gender, occupation and income group. Income level was determined by asking informants if they earned less, about the same or more than the median weekly income in Australia.^1^

### Data collection

Data were collected by six male and female research assistants with undergraduate qualifications, fluent in the language relevant to the informant. They received training in informant recruitment, the consent process and conducting interviews. Interviews were conducted as a face-to-face IDI in a quiet space after hair services or online. An IDI script was developed and used to guide the interviews (see online supplemental file 3). The IDI was audio-recorded and ranged in length from 25 to 45 min. Topics covered in the IDI guidelines included sociodemographic information, perceptions around water safety, access to water safety facilities and perceived ideas for interventions and strategies.

The English IDI interview script was translated into the following languages: simple and traditional Chinese, Hindi, Korean, Arabic and Nepali. The IDI was conducted in English or the local language of each migrant informant by a researcher who spoke the language, whichever was preferred by the informant. All interviews were translated to English for analysis. Daily debrief sessions were conducted by the lead investigators with the research assistants post data collection. No interviews were repeated. Transcripts were not returned to informants for comment due to logistical and literacy barriers.

### Patient and Public Involvement

Migrant advisors were engaged to identify hairdressers for recruitment, which themselves were often run by migrants. Migrant hairdressers provided assistance in identifying potential participants for the study.

### Ethics

All informants provided written informed consent. Ethics approval was received from the University of New South Wales Human Ethics Committee (HC230346).

### Analysis

After data collection was completed, the transcripts were reviewed by the lead authors, and potential ideas and codes were discussed and noted. The transcripts were imported to NVivo software for analysis.

The transcripts were inductively coded against informant attitudes and perceptions, and barriers and enablers to water safety programmes. As transcripts were analysed, new codes were developed under perceptions, enablers and barriers, and added to as analysis progressed. These were cross-checked against themes initially noted in debrief sessions to ensure no key points were missed. Informants were also mapped against intersectional characteristics including income, age, gender, ethnicity and occupation.

Matrix coding was used to assess similarities and differences in perceptions, enablers and barriers by intersectional groups, such as along gender lines, ethnicity and income level.

## Results

A total of 30 informants were interviewed. The demographic information of the informants is listed in table 1. Informants ranged in age from 18 to over 60 and were a mix of male and female gender. A variety of ethnicities were also interviewed, including Indian, Korean and Arab migrants. Informants were largely employed or working or students, and of various income levels.

**Table 1:** Summary of respondents

Intersectional characteristic		Number of informants
Age		
	Younger adult (18 to 39 years old)	17
	Older adult (40+years old)	4
	Unreported	9
Gender		
	Male	16
	Female	9
	Unreported	5
Ethnicity		
	Arab	3
	Bangladeshi	2
	Chinese	2
	Indian	15
	Korean	6
	Nepalese	2
Occupation		
	Employed/working	17
	Unemployed	3
	Students	11
Income		
	Below median	4
	Median	5
	Above median	9
	Unreported	12

Overall, four main themes were identified, covering current migrant access to water and water safety programs, knowledge and perceptions towards water safety, and key barriers and enablers to accessing water safety programs. These revealed a picture that migrants in the greater community lacked awareness of water safety programmes despite having some interest to learn. Other barriers identified include time constraints and availability of suitable programmes.

Table 2 lists the key enablers and barriers identified in the analysis. These are discussed in detail in the results section below.

**Table 2:** Key barriers and enablers to programme participation

Barriers	Enablers
Lack of knowledge and communication about programmes (Theme 4.1.2) Fear of aquatic life and drowning in open water (Theme 4.2.3) Lack of timely and affordable transport (Theme 4.3.1) Competing priorities and lack of time (Theme 4.3.2, Theme 4.5.4) Cost of accessing programmes (Theme 4.5.3) Perceived unimportance of learning water safety skills for those who do not access water (Theme 4.5.1)	Perceived importance of learning water safety skills (Theme 4.2.1, Theme 4.4.3) Availability of water safety signs to improve awareness of water safety risks (Theme 4.2.2) Perceived safety in enclosed water bodies such as pools (Theme 4.2.3) Presence of lifeguards at beaches and in pools (Theme 4.2.3) Social networks and community support from fellow migrants (Theme 4.4.1, Theme 4.5.1) Perception of poor swimming capability and previous drowning experiences (Theme 4.4.2) Use of regional languages in training (Theme 4.4.4) Communication of available programmes through university and community channels (Theme 4.4.5) Occupational requirements (Theme 4.4.6) Culturally sensitive programmes (Theme 4.5.5)

We also identified several differences between groups on intersectional axes, in particular age and ethnicity. These are discussed at the end of the results section.

Quotations are provided with the gender, ethnicity and age group of the informants.

### Theme 1: Access to water and water safety programmes

#### Exposure to water

Very few informants had engaged in any water-safety related courses or classes since arriving in Australia. However, the majority of informants had experienced recreational exposure to water bodies for recreational activities, though at varying degrees. Those living, working or studying closer to a beach or pool accessed water more frequently than those who lived further away.

There is an aquatic centre in Olympic Park, it’s near to my house and sometimes we go there if the weather is like summer.- ID101 (Female, Arab, 30s)

#### Awareness of water safety programmes

A major barrier to accessing water safety programmes was the lack of knowledge about the programmes and subsidised schemes available. Most informants had never heard of available water safety programmes.

“No, I have not availed any education about water safety after coming to Australia…I am not aware of any such programs.- ID10 (Female, Indian, 20s)

Only one informant had used swimming vouchers through government programmes. The informant used the vouchers for swim classes for their children, not themselves.

Yes of course I used them….The Active Kids.– ID102 (Female, Arab, 20s)

### Theme 2: Knowledge and Perceptions of Water Safety

#### Perceived importance of learning water safety skills

The majority of informants believed water safety skills were important. This was for a variety of reasons, such as belief in its importance for survival, the importance of swimming for exercise, the importance of learning rescue skills and because they enjoyed water-based activities.

Ah I love the feeling of the sea breeze and the colour of the ocean…I love blue waters and just being in nature is something I enjoy…so I think it’s something that really calms me down… – ID8 (Female, Indian, 20s)

Only a few informants stated they have no interest at all because they had limited contact with water. Some informants also mentioned that as water safety was not relevant to their income source, they were less interested in learning.

Yes, if I liked water and played water sports like surfing then I would have looked into getting more swimming lessons to improve my skills, but I didn’t really have much interest, so I felt no need to get more lessons.– ID86 (Male, Korean, 20s)

If I was working in a job related to water, like if I was a pool trainer, then maybe. But the kind of job I do and the area I live in, it’s not [relevant]. - ID11 (Male, Indian, 20s)

#### Awareness of water safety risks

Signage may be a useful method to disseminate water safety information. The majority of informants noted having seen water safety signages at water bodies, especially popular swim areas. But informants paid varying levels of attention to these signs. Most noted that signs were easily located, though some noted they could be placed in higher visibility areas like parking lots.

I have noticed sometimes signs saying, “Beware of high waves”or “Swim only between the red flags in the beach”. Apart from these, I have not really paid too much attention to the signage.- ID10 (Female, Indian, 20s)

Some informants were careful to note the signs when deciding where to enter the water. Participants stated that signs are important and generally easy to understand and should be more widely available across water bodies.

Beaches, [there are]some [signs]. The famous ones, yes. Like Bondi and Coogee will have [signs] but there are a lot of beaches here in Sydney. I'd say apart from the 2-3 famous ones, the others don't really have signage or normal swimming safety instructions, like shark safety and stuff like that.- ID9 (Female, Indian, 20s)

#### Perceptions of hazards in water environments

For many informants, fear of aquatic life, such as jellyfish and sharks, was at the forefront of their concerns regarding beach and natural environments. This was mentioned by more informants than a fear of drowning.

He had shared a story about participating in a swimming competition at Coogee and being injured, possibly by a shark. It was not far from the beach, and he had to be rescued by a medical helicopter.– ID26 (Male, Nepali, Unknown Age)

If a jellyfish comes and touches me while swimming, I may die. I'm quite afraid of jellyfish.– ID42 (Unknown gender, Malaysian, 30s)

Migrants felt safer swimming in enclosed areas such as pools and spas compared with beaches or other natural water bodies. These environments were considered safer due to less depth, still water, regulated temperature, more people present and predictability. This was especially true of informants who reported poor swimming capability.

There are a lot of differences between swimming in a pond [pool] and the ocean. The current is a huge factor here in the pond - you will not face that kind of current… Also, in the swimming pool [the depth] is kind of similar.– ID25 (Male, Bangladeshi, Unknown age)

Some migrants were aware of ocean swimming pools and noted that these may be the best of both worlds as they were free for use and provided the security of pools.

Another key factor that increased interest in water access was the availability of lifeguards. Some informants noted that in pools, the lifeguards were located closer to swimmers compared with oceans. The presence of lifeguards was considered comforting and improved their experience of water bodies. Some informants noted they were more likely to enjoy water activities and programmes if the ratio of qualified trainers to students was higher.

I didn't feel concerned… I saw there are some volunteers [lifeguards] always nearby. So, these places seem good.– ID25 (Male, Bangladeshi, Unknown age)

### Theme 3: Barriers to Participation in Water Safety Programmes

#### Accessibility

For many informants, transport was not seen as a challenge to access water bodies. Transport connectivity in Sydney was considered adequate.

…access is easy, especially because of public transport. So, whenever we had a day off, if I went with my friends, it was easy to go there.- ID7 (Male, Indian, 20s)

However, transport was a concern for some migrants living further inland or far from water facilities. For some informants of lower income, transport cost and time presented a barrier to access. For those who did not own a car, public transport was sometimes considered time-consuming.

It takes a lot of time via public transport to reach them [water bodies] and I feel like transporting by Ubers can be costly as well, especially over the weekend.- ID8 (Female, Indian, 20s)

#### Time constraints

The majority of informants noted they had limited time to participate in water safety programmes or water activities. Although informants agreed water safety was important, it was not as pressing as other priorities in Australia, such as earning an income and caretaking of children.

Our mind is always occupied with where I can get a better job that pays better. How can I get my permanent residency? We have been brought up with an understanding that we need to work hard and make a better living. Swimming and possibly some other sports are more for fun and luxury. We never felt that swimming and such activities can or should be a part of life.- ID26 (Male, Nepali, Unknown Age)

### Theme 4: Facilitators of Participation in Water Safety Programmes

#### Social networks and community support

For many informants, social connections were a major enabler in accessing water-related activities. Some informants said they first heard about water safety and swimming programmes from other members of their community.

The people you associate yourself with is how you behave…So I stayed with them, worked with them, and whatever their activities were, I went with them [to water bodies]. I found it was nice; it was fun. So, I started going on my own too.- ID11 (Male, Indian, 20s)

Many informants noted that being able to swim and access water bodies was essential to fully participate in Australian culture and build connections with Australians, given the prevalence of water in Australian culture. This was especially encouraging for children, where parents welcomed the opportunity for generational assimilation.

I think it is essential for immigrant kids in Australia to learn swimming. Other than being an essential life skill, learning how to swim is a compulsion for us to be a part of the community, especially when we have decided to live in Australia.– ID26 (Male, Nepali, Unknown Age)

#### Poor swimming capability

Largely, informants’ previous experience at home dictated their current level of skill and comfort in the water. Informants reported varying degrees of swim ability, where some possessed no knowledge of swimming, some had been taught in their local contexts via non-curriculum-based methods and a small minority had received curriculum-based training. Generally, those who had access to formal training were of higher income or lived in larger cities before moving to Australia.

Their style of teaching was also funny - he lifted me and just threw me in the lake and said go [swim] yourself using your hands… So, in the starting I would just use my arms, but with time it came to me. There was no specific training.- ID11 (Male, Indian, 20s)

One barrier for informants was the belief that learning as an adult would be difficult.

It’s best to learn swimming as a child. When you become an adult, you are more scared and it’s difficult to gather all the courage needed to swim.- ID26 (Male, Nepali, Unknown Age)

For some informants, previous scares with near-drownings, or hearing stories about drowning events among their acquaintances, increased reluctance to enter the water. For others, it was a reason to learn to swim.

But after that [incident] he is also traumatised. But also in the good way, he wants to learn how to swim.- ID101 (Female, Arab, 30s)

Of those who self-reported poor or inadequate swimming skills, many displayed an interest in learning swimming and water safety. Many also noted a fear of drowning. For some, poor swimming ability was a reason to learn formally, while others found that fear prevented them from attempting a water safety programme.

Yeah, possibly a tsunami or some cyclone or something might happen. We don't know. So always good to learn it.- ID22 (Male, Indian, Unknown age)

I would say that I'm always a little scared about the risk of drowning because I have not done a very specialised course in water safety.- ID10 (Female, Indian, 20s)

Informants who believed they had adequate swimming skills were less interested in attending water safety programmes, but some were still interested in taking CPR training.

#### Generational interest

While they themselves had not participated in water safety programmes, the majority of parents prioritised their children’s learning, many enrolling their children into classes even if they were of median or lower income status. Accessibility of classes in schools was an enabler of this. Some enrolled their children into extra classes outside of school, and a few accessed affordable or free classes given in school programmes.

I signed up my son from when he was 6 months old in swimming classes here. They told me he can’t learn how to swim at this age, but at least he can help himself get up if he might drown, God forbid.– ID105 (Female, Arab, 30s)

“Yes, it’s expensive, but because they are kids and there’s activities for kids it’s okay for me to pay for small activities. But to be honest, to pay for both me and them is too much.- ID102 (Female, Arab, 20s)

#### Language of programmes

Although not strictly required, some informants noted that using regional languages may be helpful in encouraging migrants to attend water safety classes. They may feel more comfortable in the presence of others from their community.

And sometimes the phrases that one uses to explain things on these safety matters are better understood when they are being communicated in your own language.- ID10 (Female, Indian, 20s)

#### Communication and timing of programmes

Informants provided suggestions for timing programme communications to 1 month to 1–2 years after migration. The reasoning provided was that swim safety could only be a priority after the migrants settled into their new lives by finding a home and a job. After these pressing issues were taken care of, there may be time and bandwidth to focus on water safety.

I think after they settle down and everything is going okay with them- when they already know what they want to do. They have to become stable at work, make their home stable. They won’t be doing any other activity because it’s not 100% urgent. It’s something extra.- ID101 (Female, Arab, 30s)

Some informants mentioned that earlier training should be considered as new migrants may start accessing water bodies in the excitement of exploring. However, informants noted that these migrants may not prioritise going to formal programmes, so online material to teach some basic water safety knowledge before arrival to Australia would be helpful.

“Before coming they should know a little bit. Should know a little swimming too. Because in India you get the time to [learn], but once you are here you don’t get the time. Because of work and studies, you get busy.- ID5 (Male, Indian, 20s)

Informants noted that community magazines could be used to disseminate information on water safety programmes.

Well, Korean community magazines print articles on drowning, but rather than leaving it at that, it will be useful to include information about water safety programs.- ID86 (Male, Korean, 20s)

One informant also suggested that lifeguards at popular beaches can conduct spontaneous information sessions at the beach or water locations where migrants tend to be present. Universities and schools were also considered good mediums for disseminating information.

But say if I went to the beach and these programs are being conducted out in the open in which lifeguards are demonstrating CPR steps, wouldn’t people stand around and watch and learn a few things?- ID87 (Female, Korean, 50s)

#### Occupational requirements

Formal work or course requirements acted as an enabler to learning water safety practices. CPR training was only taken by those who required the qualifications for university courses or employment. These trainings were not drowning or water-safety specific but incorporated some elements that could be transferred to water safety.

[Drowning rescue] training? No, not really, because I am studying. But recently I have studied individual sport in which I did a certificate in aged care. In that I learnt CPR and other skills because it has [relevance to] jobs in the medical field later.– ID4 (Male, Indian, 20s)

### Findings through an intersectional lens

While many perceptions, barriers and enablers towards water safety programmes were shared across migrant groups, some key differences in experiences were identified across intersectional lines by age, gender, ethnicity, income level and occupation.

#### Age

Differences in perception by age were a recurring theme through informant interviews.

While informants noted that younger migrants may be interested in joining water safety programmes so they can try new activities, older informants displayed greater reluctance. Older, employed informants also noted that there were few classes available at timeslots suitable for them. Many of the classes available were catering to children and were not timed to be accessible post-working hours.

Wherever the sea is, it’s totally free, so I just want to have a kind of fearless stage that we should try all these water bodies like water games…– ID3 (Male, Indian, 20s)

There are no programs for adults. If there are lessons for adults who go to work once or twice a week, even just once a week, it will be great. I want programs in the evening, but they are mostly for children. All the adult programs including for seniors are held in the mornings. I wish there were swimming lessons for adult learners who go to work, after 7pm.- ID87 (Female, Korean, 50s)

While for younger informants, social connection with friends was an enabler for participation, for older informants, spending time with family was important. For those who did participate in water activities, they found it provided a good opportunity to bond with family.

[Our son has] weekly lessons every week and it’s a nice activity with the family. He goes with his dad, it’s like bonding.-ID105 (Female, Arab, 30s)

#### Gender

Men in particular stated that swimming was important for exercise. Sports and fitness were associated with interest in water safety training.

Because from my perspective, swimming is a great exercise medium. So, if you do swimming regularly then you will be fitter, and you don't need to walk that much.– ID25 (Male, Bangladeshi, Unknown age)

#### Income level

Disparities in access to water safety programmes were identified along income lines. Employed and high-income informants appeared to have greater access to safer environments, such as aquatic centres and private pools, so they were more comfortable to access water bodies for lessons or training.

Oh, so the previous apartment where I used to live had a swimming pool, so we used to go often to swim…As I know swimming so it’s never [been a] hard thing for me.– ID103 (Female, Bangladeshi, 30s)

Many informants expressed that the cost of swim lessons may be a concern, especially some low and median-income informants. They stated that vouchers or subsidies would encourage more migrants to participate.

People don’t easily volunteer their time. I think it will be difficult to get people to voluntarily join these programs unless there are vouchers.- ID85 (Male, Korean, 50s)

I have enquired about swimming lessons in two or three places, but the cost of learning swimming is very expensive, especially for immigrants like me. So, that also affects [me] a bit.- ID7 (Male, Indian, 20s)

However, some above-average and median-income informants noted that paying for swim safety programmes was a worthwhile investment.

No, that [the cost] is okay. Per head the cost is $100 or $120. They provide around 10 days training… But I feel it is not too costly, still bearable.- ID22 (Male, Indian, Unknown age)

#### Occupation

Key differences in interest, access and availability were identified byween students and employed informants.

Overall, university environments allowed greater access to water safety programmes for students. Some students discussed the easy availability of pool facilities and programmes on campus. Students may be more likely to have access to information for water safety programmes through their universities, compared with those who are working.

I've heard they do swim training in UNSW every semester break or so. But yeah, I've never been.- ID23 (Unknown Gender, Indian, Unknown Age)

One informant noted that while there were swim training programmes available in their university, they booked out quickly. This suggested high demand for water safety training among students.

[There are] free courses and lessons for people who don’t know [how to swim] from Sydney University. But they have to put their name [down], and they will only select a certain number.- ID101 (Female, Arab, 30s)

Students were also more likely to be encouraged by their mentors to live a balanced life, so found the time to invest in recreational activities and enabling access to water activities.

It was in my first year of being a PhD [student]… Even my supervisors were supportive of work life balance and suggested to me to doing something extra.- ID26 (Male, Nepali, Unknown Age)

The impact of social connections on access to water activities was especially strong for students. Participation in water activities allowed many students to develop friendships, and many were first introduced to these activities through friends. Some students who were unable to swim stated that their socialisation was impacted.

If any friend anytime says let’s go to the beach, the main problem is swimming. You have to straight up say, I can’t swim, I can’t go. So yes, it does have an impact [on friendships].- ID2 (Male, Indian, Unknown Age)

Students may be introduced to water safety programmes earlier than employed migrants, as there is less academic pressure at university the first few months.

I think the most optimal time would be within a month of their arrival. I think it is in the first two or three months that one is very excited about visiting places and learning new things…And I think the pressure of academics is also not as high in the initial first month.- ID10 (Female, Indian, 20s)

#### Ethnicity

Ethnic differences in perceptions and experiences were identified.

Some female informants of Arab and Bangladeshi origin noted that women may be more comfortable in women-only classes. One informant observed that hijab-wearing women may be made to feel uncomfortable at public pools, but the informant herself had not experienced any racism.

But I hear some stories from my friend that some people here hate people wearing a hijab in the pool. [They say] ‘Why are you wearing hijab in the pool? ‘Why are you coming in if you are wearing that?’. - ID102 (Female, Arab, 20s)

Ethnic differences in the role of social connections were also clear. Some ethnicities may be less likely to socialise around water if water activities were not an important part of their culture. For example, some Nepalese informants noted that they were not exposed to water-based activities as their friends and family preferred to socialise in other settings.

No, not going to beach doesn’t reduce my social connections. Like I said, we have many festivals that we celebrate in my country. So, socializingfor us happens more during events organised for the festivals.– ID21 (Female, Nepali, Unknown Age)

The types of water activity engaged in were also heavily influenced by cultural norms. For example, Korean and Chinese informants discussed their frequent involvement in cliff fishing and regularly accessing coastlines on low-level cliffs to socialise. Korean and Chinese informants noted that programmes should consider including water safety aspects related to ocean fishing to make them more relevant.

I think Koreans are not very well informed about the dangers of high waves on cliffs. I had once experienced high waves and had all my fishing equipment swept away. It’s very hard to know if no one tells you or warns you and you are exposed to danger.- ID85 (Male, Korean, 50s)

## Discussion

This study aimed to identify barriers and enablers faced by migrants in a high-income context in Australia when accessing water safety programmes. To better generalise our findings to the needs of migrant populations, we approached informants across linguistically diverse groups in a non-water-based setting, hairdressing salons. This enabled us to identify programme design opportunities to improve participation, while avoiding the homogenisation of migrant experiences. The results demonstrated a significant lack of programme reach and awareness and key barriers faced by migrants. We also identified some important differences in perceptions between group intersections that may be taken into consideration when designing and delivering programmes to improve uptake.

The most significant result found was that the majority of informants had very low awareness of water safety programmes available in Australia. Only some university students were aware of swim training available in their campuses, and one employed informant had used a voucher subsidy scheme for their children. Improved outreach to migrants is crucial to inform migrants of available programmes in their communities. Informants suggested collaborating with community-based magazines and university notification channels to inform migrants of opportunities. Schools could also be used to target migrant parents, pitching water safety programmes as a way to both improve swimming skills and participate in family bonding time.^10^

Interestingly, our research found that migrants were aware of water safety signage and messaging around water bodies, contrary to previous research.^11^ However, the extent to which these messages are correctly understood remains uncertain and warrants further investigation. Consequently, signage may represent a promising avenue for effectively communicating key information about water safety programmes.^12^ Multisectoral partnerships are also crucial to address drowning risks,^13^ where the Immigration Department may also consider sharing water safety and programme information with incoming migrants before they arrive in Australia, and local municipalities and Department of Education may support implementation of programmes through community pools, schools and universities.^14^

The timing of communication to migrants was also considered important. Our study found that communication should be targeted to migrants when they have the capacity to consider participation after settling into their new lives. Multiple touchpoints could be considered between the first month of arrival to about 2 years after arrival. Students, in particular, could be engaged earlier before academic pressure increased at university. Potential touchpoints for engaging migrant populations could include English competency examinations, such as IELTS or TOEFL, which are commonly undertaken as part of visa requirements. Partnerships with local council programmes focused on integration and language development may also provide effective avenues for outreach. Furthermore, leveraging co-benefits associated with health promotion programmes could be advantageous, particularly for addressing non-communicable diseases (NCDs) among older adults within migrant populations, who are at a heightened risk for such conditions. The use of WhatsApp forwards has also been successful for recruiting migrants for research and could be employed for programmes.^15^ This would allow multiple points of communication at different times. Word-of-mouth has additionally been shown to be a powerful tool, where programme informants may be incentivised to enrol other migrants in their community who are ready to participate.^16^

The content of communication should be tailored to the priorities of the target groups.^17^ In our study, we found that focussing on the relevance of water safety knowledge to fishing may encourage participation of Korean and Chinese migrants. When targeting men, messaging may focus on the sport and fitness aspect of swimming. Promotions aiming to raise programme awareness among migrants could emphasise the importance of water safety skills as a pathway to enjoying Australian culture and lifestyle, contributing to social cohesion, employment outcomes and youth engagement, a suggestion that has been made in other studies of migrant perceptions.^18^ To encourage those who have a fear of drowning, messaging could also focus on the methods used by practitioners to ensure a safe swim environment and cultural sensitivity during classes.^19 20^

Differences in experiences by age were a key intersectional finding. While a high demand in student populations for water safety training was indicated, older adults faced considerable challenges in prioritisation and availability. While most parents ensured their children learnt swimming, many did not prioritise their own learning due to time, cost or availability constraints. Accessible, affordable programmes, held at convenient times post-working hours, are required to address the needs of older migrants. It is also possible that communication about programmes was not reaching older migrants, who are less connected to established communities like universities or clubs. Unfortunately, there is limited research from other high-income contexts exploring how older adults can be encouraged to participate in water safety programmes. However, research on adult participation in recreational physical activity has found that the creation of activity-based social groups and encouragement from spiritual and community leaders may improve attendance.^21^,^23^ While state-level Education Departments in Australia incorporate water safety training in school curriculums for children, policies and subsidised programmes aiming to facilitate swimming training in adult migrants are relatively emergent. Practitioners and government both can benefit from developing programmes specifically targeting working adults.

Our findings further demonstrated that programmes should be available in aquatic centres, considered safer environments than natural water bodies by migrants. However, our results showed that the recurring cost of using these facilities may become a deterrent. Migrant integration policies can consider improving accessibility, such as by subsidising the costs of visiting aquatic centres for the first few months after migrant settlement. Current programmes are inadequate for such provision or encouragement.

Cultural and gender differences may also play a role in how accessible programmes are. Arab women, in particular, may require women-only classes. Korean and Chinese migrants may be more interested in attending programmes if there is guidance on cliff safety, given their greater participation in fishing activities. Trainers who can deliver the training in migrants’ mother tongues may also encourage greater interest. A literature review of studies evaluating global water safety programmes in high-income contexts recommended that practitioners develop programmes for minorities with members of the target group to ensure translations and approaches are culturally relevant and appropriate for understanding.^24^ This approach has found success in some Australian swim training programmes which were modified to meet cultural requirements of Afghan migrants, who preferred gender-segregated classes.^25^

Our results, however, demonstrate that improved communication and availability of suitable programmes may not be sufficient to ensure participation of migrant populations. While most of the informants indicated interest in learning water safety skills, the majority emphasised the low priority of learning water safety compared with other pressing matters, such as income generation and studying. Reducing the cost of programmes may encourage participation, as well as leveraging social connections, especially for students. However, innovative methods are required to encourage the participation of older migrants and migrants from cultures that place less emphasis on water activities. For example, an African migrant support programme in Australia established community gardens and encouraged local low-income migrants to participate in workshops and gardening, enhancing well-being.^26^ A similar approach could be applied for water safety programmes, where social groups of different ethnicities can be organised to attend training together to build connectedness.

A workshop convened by Royal Life Saving Australia on behalf of the Australian Water Safety Council recommended fostering cross-sectoral partnerships among water safety organisations, swim programme providers, multicultural agencies and community leaders to drive community level change.^27^ The importance of the settlement phase for refugees and migrants, where efforts to build resilience and safety around water, and realise the health, social and economic benefits of aquatics, needs to be acknowledged and resourced at the highest levels of decision-making.^28^ Overall, this study highlighted key gaps in migrant participation in water safety programmes. Practitioners should seek to improve communication and outreach throughout the first 2 years of migration. Practitioners may also improve participation by catering to the needs of older migrants and certain ethnicities. However, this cannot be achieved without policy support and multisectoral partnerships with local councils and government departments such as Education and Immigration who can assist with resources and can provide information and access to migrant populations.^13^

### Limitations of the study

While a choice was given to informants to speak in their native language, most informants opted to speak in English. However, we are uncertain of the fluency of English spoken. In addition, due to issues with understanding and translation, some leading questions were used as informants did not have enough background knowledge to understand some of the questions being asked. This was partly mitigated by repeating informants’ opinions back to them and asking them to revise if incorrect.

Another potential limitation of the study lay in the selection of hairdresser salons as points of engagement with migrant community members outside water-related activity settings. While this approach facilitated access to certain segments of the population, it may not comprehensively reach individuals across all economic strata. Alternative venues, such as religious organisations or local grocery stores catering to specific ethnic groups, might provide more inclusive access to a broader cross-section of the community.

Our study identified some intersectional differences in perceptions of water safety programmes based on factors such as age, ethnicity, occupation and income. However, deeper analysis of these nuances proved challenging due to the complexity of sub-groups along intersecting axes, such as gender and ethnicity or income and occupation. Addressing these gaps will require a larger sample with sufficient representation across sub-groups to identify meaningful differences.^29^ Future quantitative and qualitative research should aim to conceptualise intersectional groups with the lowest likelihood of participating in water safety programmes.

## Conclusion

Drowning disproportionately affects migrant populations, highlighting the urgent need for inclusive and culturally sensitive water safety interventions. By employing community-based recruitment and an intersectional approach, this research provided a nuanced perspective on how factors such as ethnicity, gender, income and occupation shape migrants’ engagement with these programmes. The findings emphasised that addressing systemic inequities and cultural barriers is critical for designing effective, inclusive strategies that ensure water safety for all. Moving forward, policies and programmes must be co-designed with migrant communities to prioritise accessibility, cultural relevance and community-driven solutions, thereby reducing drowning risks and promoting broader water safety awareness. This approach is essential not only for Australia but also for other countries grappling with similar challenges among diverse and multicultural communities.

## Supplementary material

10.1136/bmjopen-2025-107233 online supplemental file 1

## Data Availability

Data are available upon reasonable request by contacting the lead author.
