# Supplementary material for: Qualitative exploration of barriers and enablers to migrant access to water safety programmes in Australia
Source: BMJ Open. 2025 Nov 4;15(11):e107233. doi: 10.1136/bmjopen-2025-107233 (PMC12587953; doi:10.1136/bmjopen-2025-107233)
Supplement: online supplemental file 1 [file bmjopen-15-11-s001.docx]

**Supplementary Files**

**Supplementary File 1 – Original protocol for the study**

**
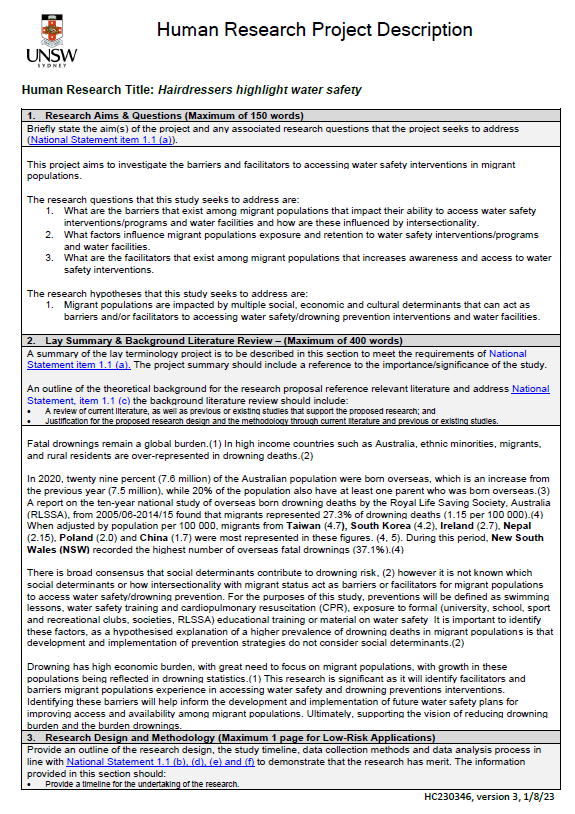
**

**
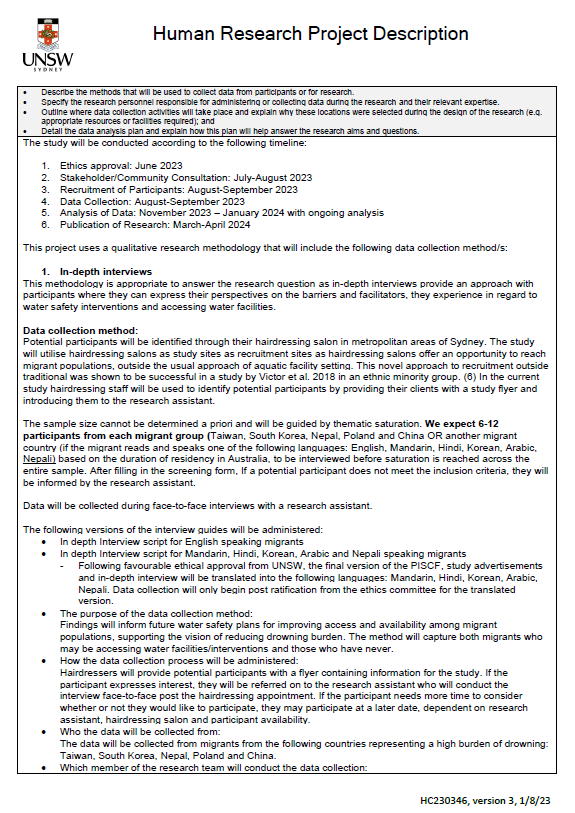
**

**
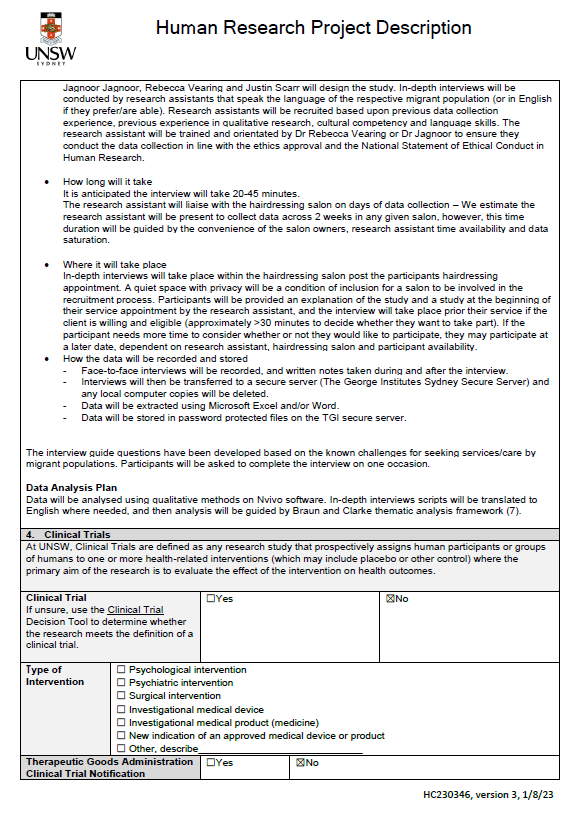
**

**
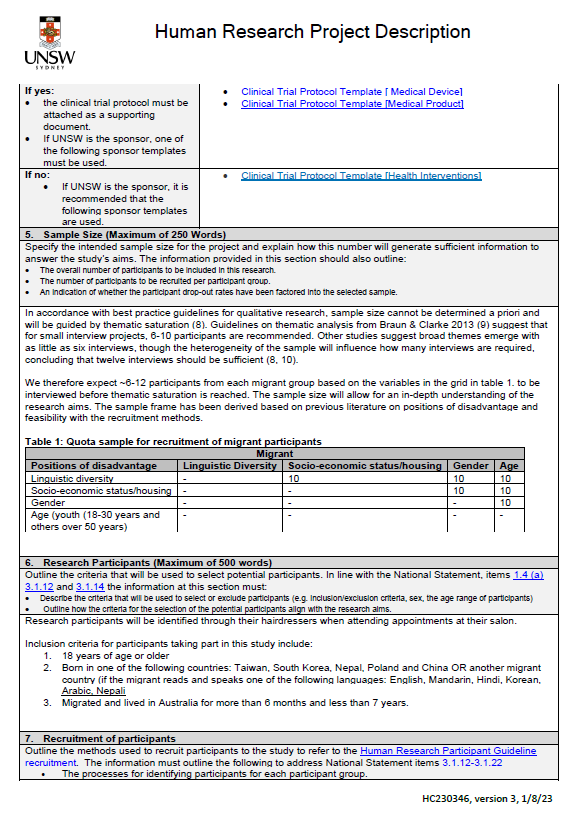
**

**
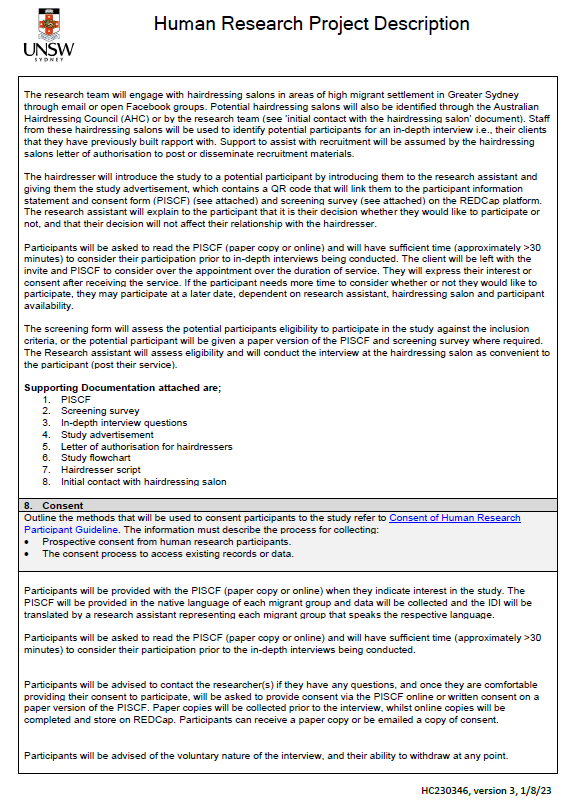
**

**
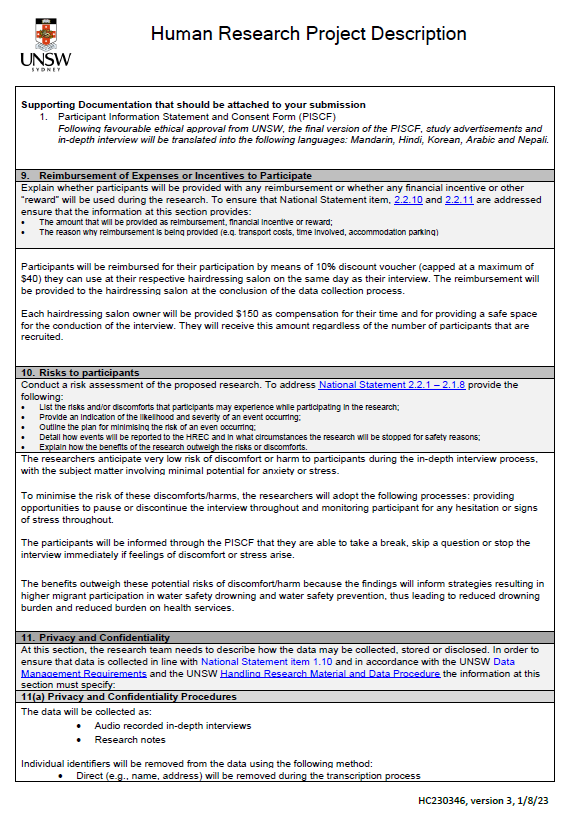
**

**
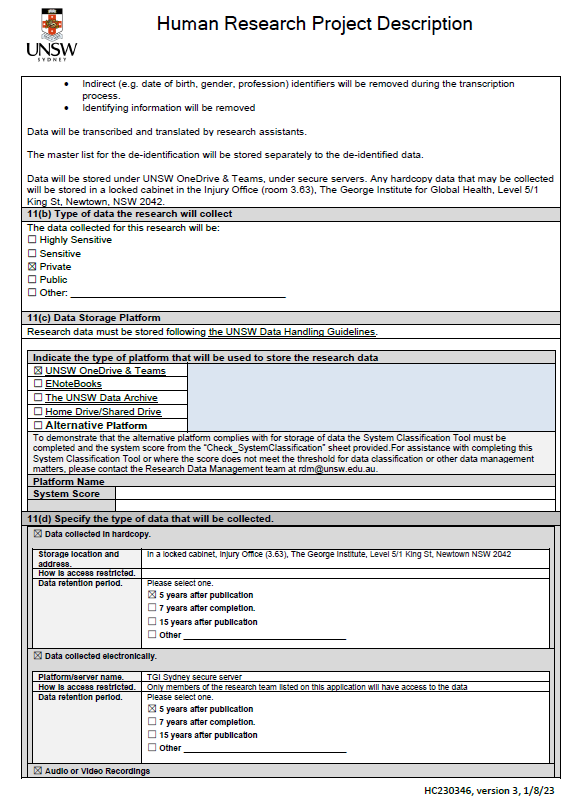
**

**
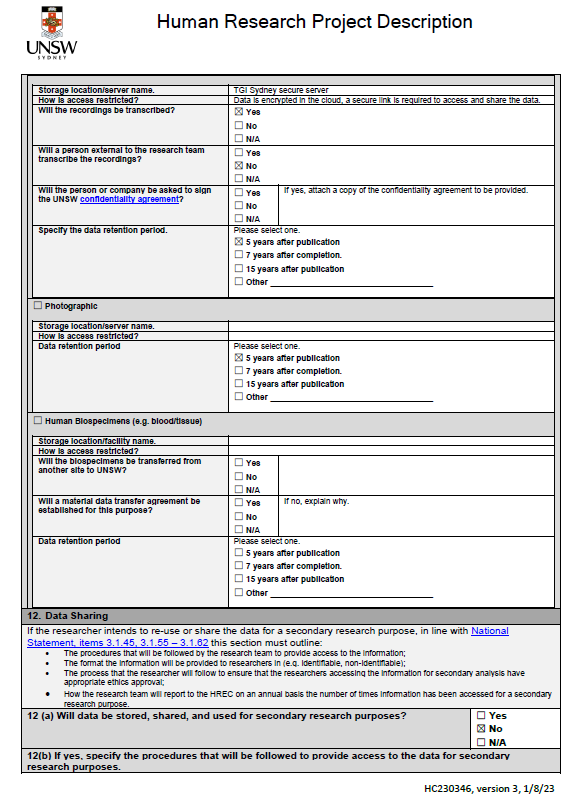
**

**
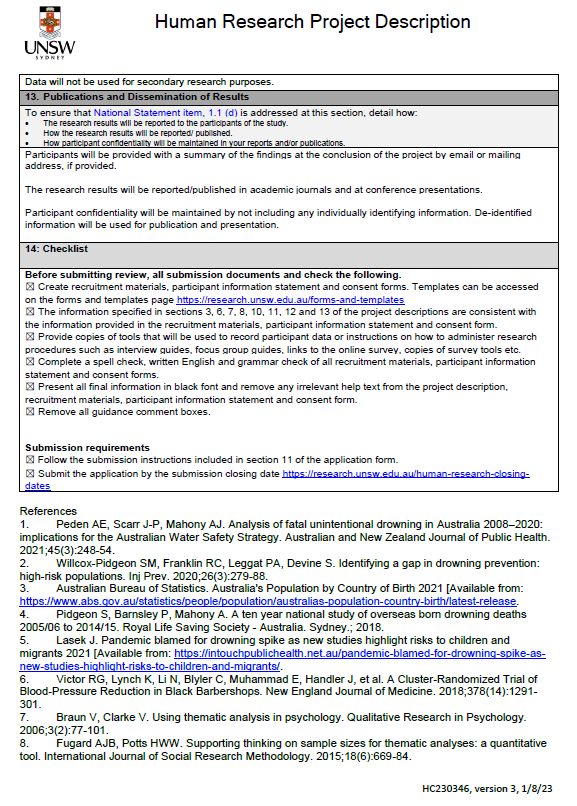
**

**
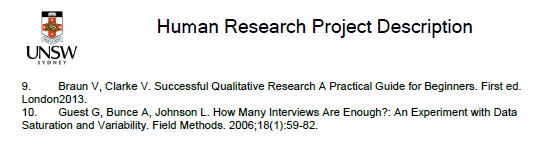
**

**Supplementary File 2 - List of suburbs in Sydney where hairdressing salons were identified**

1. Chatswood
2. Eastwood
3. Harris Park
4. Kogarah
5. Hurstville
6. Macquire Park
7. Granville
8. Merrylands

**Supplementary File 3 – In-depth Interview guide**

**In depth interview Fact-sheet**

Participant number |__|__|__|__|           Facilitator Initials |__|__|__|                Note-taker Initials |__|__|__|

Audio file: |__|__|__|       Date   |__|__/__|__/__|__|

Introduction

I am ______________________________ from The George Institute and I will conduct this interview (facilitator) and I will be taking notes (note-taker)

⎫ Introduce yourself to the respondent (i.e. the client)

⎫ If the client is not familiar to you spend a few minutes talking with them. Please clearly make them understand what you want to do and explain the audio recording of interview, the need as per informed consent form.

⎫ Let the client know you are going to conduct an interview with the respondent/client and seek permission to conduct interview in a quiet place and humbly tell them you would appreciate no interruptions during the interview

⎫ Make clear that if they have more queries, you are happy to further discuss and explain the research work

⎫ Make sure a quiet place is available for interview (to avoid interruption and make sure clear voice recording),

⎫ Firstly, you can start with some informal conversation with respondent to develop rapport

⎫ Importantly, make sure respondent are comfortable and a good rapport has been built to ensure they are able to share their beliefs and experiences

⎫ You will explain the study while arranging IDI, however you must re-introduce the research area and information sought.

⎫ Please ensure informed online consent (on REDCap) or written consent is obtained from the participant

⎫ Clearly explain them why you are using audio recording. Explain why audio recording is needed and that the information is de- identified for analysis. Reiterate that their participation is voluntary and they can withdraw consent at any time, any data collected/ recording made will be deleted immediately.

Record relevant demographic information- age, gender, place, occupation and assign a ID.

⎫        Please check the recorder is working. Ensure you have spare batteries as needed. Check on recorder if possible during the interview and certainly on completion.

⎫ All interviews are to be conducted face to face

⎫ Make note of body language and expressions as relevant

⎫ After completion of interview, thank the participant for their time. Check on recording before leaving the premises.

*** Soon after completing the interview, fill in the interview environment sheet***

**Interview environment sheet**

*(To be filled in post interview – notes may be taken as relevant during the interview)*

1. Please describe how you select your respondent, (write down everything, sampling, rapport build up everything)

2. Please describe the place you have taken interview (details)

3. Describe any interruption during interview

4. How was the respondent during the interview? Did they interact well, if not then why?

5. Did they have any expression on particular issue? (please describe details)

**Prompts sheet**

1. How long have you been in Australia for since migration?
2. What is your occupation?
3. How would you describe your swimming capability?
   1. Where did you learn? How were you taught?
   2. Why did you learn?
   3. What have you not learned how to swim?
4. Do you access water bodies in Australia, for example beaches and pools?
   1. Which ones?
   2. Why do you access the water bodies?
   3. Who do you go with?
   4. What do you do in at the water bodies?
   5. How frequently do you access the water bodies?
   6. If you rarely access the water bodies, why is that?
5. Have you participated in any water safety programs, e.g. Active Kids, Classes in your local acquatic centre?
   1. Why or why not?
6. Have you heard of any water safety programs since arriving to Australia?
   1. If yes, which have you heard of?
7. How do you think these programs could best inform to you? What
8. Would you consider participating in any water safety programs?
   1. Why or why not?
   2. What would make you more interested in participating?
9. How important do you think water safety programs are?
   1. Why are they important, or why not?
10. *And have you seen any water safety signs at water bodies at all?*
    1. *What do they say usually?*
    2. *Do you pay attention? Why or why not?*
11. *What do you think about the cost of water safety programs?*
12. *How easy is it to access water bodies?*
    1. *What makes them easy to access?*
    2. *What makes them hard to access?*
13. If more of your friends or family access water bodies, would you also go?
    1. Why or why not?
    2. What if they attended the water safety programs with you?
14. After migration to Australia, when do you think is the best time to participate in water safety programs?
    1. Why this timeframe?
15. Does having an instructor teach in your native language make a difference, or it doesn’t matter?
    1. Why or why not?
16. Does anyone else in your family swim or access water bodies? Tell me their activities.

**Supplementary File 4 – COREQ Checklist**

**Consolidated criteria for reporting qualitative studies (COREQ): 32-item checklist**

Developed from:

Tong A, Sainsbury P, Craig J. Consolidated criteria for reporting qualitative research (COREQ): a 32-item checklist for interviews and focus groups. *International Journal for Quality in Health Care*. 2007. Volume 19, Number 6: pp. 349 – 357

| **No. Item** | **Guide questions/description** | **Reported on Page #** |
| --- | --- | --- |
| **Domain 1: Research team and reﬂexivity** |  |  |
| *Personal Characteristics* |  |  |
| 1. Inter viewer/facilitator | Which author/s conducted the inter view or focus group? | Page 4 |
| 2. Credentials | What were the researcher’s credentials? E.g. PhD, MD | Page 4 |
| 3. Occupation | What was their occupation at the time of the study? | Page 4 |
| 4. Gender | Was the researcher male or female? | Page 4 |
| 5. Experience and training | What experience or training did the researcher have? | Page 4 |
| *Relationship with participants* |  |  |
| 6. Relationship established | Was a relationship established prior to study commencement? | Page 4  . |
| 7. Participant knowledge of the interviewer | What did the participants know about the researcher? e.g. personal goals, reasons for doing the research | Page 4 |
| 8. Interviewer characteristics | What characteristics were reported about the inter viewer/facilitator? e.g. Bias, assumptions, reasons and interests in the research topic | Page 4 |

| **Domain 2: study design** |  |  |
| --- | --- | --- |
| *Theoretical framework* |  |  |
| 9. Methodological orientation and Theory | What methodological orientation was stated to underpin the study? e.g. grounded theory, discourse analysis, ethnography, phenomenology, content analysis | Page 4 |
| *Participant selection* |  |  |
| 10. Sampling | How were participants selected? e.g. purposive, convenience, consecutive, snowball | Page 3 and 4 |
| 11. Method of approach | How were participants approached? e.g. face-to-face, telephone, mail, email | Page 3 |
| 12. Sample size | How many participants were in the study? | Page 3 |
| 13. Non-participation | How many people refused to participate or dropped out? Reasons? | Page 3 and 4 |
| *Setting* |  |  |
| 14. Setting of data collection | Where was the data collected? e.g. home, clinic, workplace | Page 3  . |
| 15. Presence of non-participants | Was anyone else present besides the participants and researchers? | Page 3 |
| 16. Description of sample | What are the important characteristics of the sample? e.g. demographic data, date | Page 5 and 5 |
| *Data collection* |  |  |
| 17. Interview guide | Were questions, prompts, guides provided by the authors? Was it pilot tested? | Page 4, Supplementary 3 |
| 18. Repeat interviews | Were repeat inter views carried out? If yes, how many? | Page 4 |
| 19. Audio/visual recording | Did the research use audio or visual recording to collect the data? | Page 4 |
| 20. Field notes | Were ﬁeld notes made during and/or after the inter view or focus group? | Page 4 |
| 21. Duration | What was the duration of the inter views or focus group? | Page 4 |
| 22. Data saturation | Was data saturation discussed? | Page 4 |
| 23. Transcripts returned | Were transcripts returned to participants for comment and/or correction? | Page 4 |
| **Domain 3: analysis and ﬁndings** |  |  |
| *Data analysis* |  |  |
| 24. Number of data coders | How many data coders coded the data? | Page 4 |
| 25. Description of the coding tree | Did authors provide a description of the coding tree? | Page 4 |
| 26. Derivation of themes | Were themes identiﬁed in advance or derived from the data? | Page 4 |
| 27. Software | What software, if applicable, was used to manage the data? | Page 4 |
| 28. Participant checking | Did participants provide feedback on the ﬁndings? | Page 4 |
| *Reporting* |  |  |
| 29. Quotations presented | Were participant quotations presented to illustrate the themes/ﬁndings? Was each quotation identiﬁed? e.g. participant number | Page 5 to 13 |
| 30. Data and ﬁndings consistent | Was there consistency between the data presented and the ﬁndings? | Page 5 to 13 |
| 31. Clarity of major themes | Were major themes clearly presented in the ﬁndings? | Page 5 to 13 |
| 32. Clarity of minor themes | Is there a description of diverse cases or discussion of minor themes? | Page 5 to 15 |
