# Supplementary material for: Qualitative exploration of barriers and enablers to migrant access to water safety programmes in Australia
Source: BMJ Open. 2025 Nov 4;15(11):e107233. doi: 10.1136/bmjopen-2025-107233 (PMC12587953; doi:10.1136/bmjopen-2025-107233)
Supplement: Supplementary file 2 [file bmjopen-2025-107233.reviewer_comments.pdf]

PEER REVIEW HISTORY

BMJ Open publishes all reviews undertaken for accepted manuscripts. Reviewers are asked to complete a checklist review form and are provided with free text boxes to elaborate on their assessment. These free text comments are reproduced below.

ARTICLE DETAILS

Title (Provisional)

A qualitative exploration of barriers and enablers to migrant access of water safety programs in Australia

Authors

Jagnoor, Jagnoor; Scarr, Justin; Gupta, Medhavi

VERSION 1 - REVIEW

**Reviewer**	**1**
**Name**	**Beerman, Stephen**
**Affiliation**	**University of British Columbia Clinical Associate Professor and Department of Family Practice, Site Director., Dept of family practice**
**Date**	**27-Aug-2025**
**COI**	**None**

Title: This is good - Novel

Abstract: This is well done

Keywords: These are OK,

Strengths and limitations of this study - read with great interest. The denominator issue - who is at risk - all population or those who go to water areas and or put themselves at risk. This novel approach to seek information from non-aquatic environments to seek opinions/data. Asking the question - do you access water (on or in or near) with intent or without intent may have been interesting. Some of the people in non-aquatic settings may never go near water risks. Some of the people in aquatic setting may place themselves at risk to varying degrees (rare and low risk vs frequent and higher risk)

**Reviewer**	**2**
**Name**	**Guevarra, Jonathan**

Affiliation **University of the Philippines Manila, Department of Health Promotion and Education, College of Public Health, University of the Philippines Manila**

Date **28-Aug-2025**

COI **None**

Abstract

1. Include information about the specific qualitative design (in-depth interview is the technique used in collecting data and not a qualitative study design) , sampling technique, criteria for selection, method of analysis

2. Suggestion to use the term “informants” instead of participants for in-depth interviews. Please apply in the whole manuscript.

Methodology

3.1 Setting

• Focus only on the setting.

• The second paragraph (lines 37 to 40) can be included in other subsections of methodology (Participants).

3.2. Study Design

• What specific type of qualitative study design was used in this study (in-depth interview is the technique used in collecting data and not a qualitative study design)?

• The second paragraph (lines 49 to 51) can be included in other subsections of methodology (Participants).

3.5 Analysis

• Describe in detail how you conducted the Thematic Analysis

4. Results

• Lines 5-6: Give brief description of the contents/highlights of Table 1.

• What are the themes that fall under barriers? enablers? It would be good to present the themes for barriers and enablers in a matrix/table before discussing the result for each barrier/enabler.

• Include only the quotes/representative passages from the informants (for the specific identified theme) and exclude the question asked by the interviewer

5. Discussion

• Lines 14-16: Cite published article/s about multisectoral partnerships applicable in this study

• Lines 37-45: Cite literature supporting the statements/discussion written in this paragraph

---

VERSION 1 - AUTHOR RESPONSE

<u>Reviewer: 1</u>

Comments to the Author:

Title: This is good - Novel

Abstract: This is well done

Keywords: These are OK,

Strengths and limitations of this study - read with great interest. The denominator issue - who is at risk - all population or those who go to water areas and or put themselves at risk. This novel approach to seek information from non-aquatic environments to seek opinions/data.

Thank you for your positive comments.

Asking the question - do you access water (on or in or near) with intent or without intent may have been interesting. Some of the people in non-aquatic settings may never go near water risks. Some of the people in aquatic setting may place themselves at risk to varying degrees (rare and low risk vs frequent and higher risk)

We agree with the observation, and that exposure is hard to measure. This is an interesting suggestion, and we will be sure to incorporate it in future research through the lens of intersectionality.

<u>Reviewer: 2</u>

Abstract

1. Include information about the specific qualitative design (in-depth interview is the technique used in collecting data and not a qualitative study design) , sampling technique, criteria for selection, method of analysis

Keeping in mind the 250-word limit on the abstract, we have updated the methods as follows:

“**Methods:** This phenomenological qualitative study addresses these gaps by employing a novel approach to recruitment: conducting in-depth interviews with purposively-selected recent migrants in a non-traditional setting - hairdressing salons - to capture diverse perspectives. A variety of ethnicities, ages and genders were recruited. Thematic analysis with an inductive approach was used to analyse the data.”

2. Suggestion to use the term "informants" instead of participants for in-depth interviews. Please apply in the whole manuscript.

This has been done.

Methodology
3.1 Setting
• Focus only on the setting.
• The second paragraph (lines 37 to 40) can be included in other subsections of methodology (Participants).

This has been moved.

3.2. Study Design
• What specific type of qualitative study design was used in this study (in-depth interview is the technique used in collecting data and not a qualitative study design)?

The following has been added under Study Design.

"The study applied a phenomenological approach to capture informant lived experienced."

• The second paragraph (lines 49 to 51) can be included in other subsections of methodology (Participants).

This has been moved.

3.5 Analysis
• Describe in detail how you conducted the Thematic Analysis

The thematic analysis was described in greater detail as follows:

"The transcripts were inductively coded against informant attitudes and perceptions and barriers and enablers to water safety programs. As transcripts were analysed, new codes were developed under perceptions, enablers and barriers, and added to as analysis progressed. These were cross-checked against themes initially noted in debrief sessions to ensure no key points were missed. Informants were also mapped against intersectional characteristics including income, age, gender, ethnicity and occupation.

Matrix coding was used to assess similarities and differences in perceptions, enablers and barriers by intersectional groups, such as along gender lines, ethnicity and income level."

4. Results

• Lines 5-6: Give brief description of the contents/highlights of Table 1.

The following description as been added:

"Informants ranged in age from 18 to over 60, and were a mix of male and female gender. A variety of ethnicities were also interviewed, including Indian, Korean and Arab migrants. Informants were largely employed/working or students, and of various income levels."

• What are the themes that fall under barriers? enablers? It would be good to present the themes for barriers and enablers in a matrix/table before discussing the result for each barrier/enabler.

The following was added:

"Table 2 summarises the key enablers and barriers identified in the analysis and the themes where these are discussion in detail below.

Table 2: Summary of barriers and enablers to program participation

Barriers	Enablers
• Lack of knowledge and communication about programs (Theme 4.1.2)<br>• Fear of aquatic life and drowning in open water (Theme 4.2.3)<br>• Lack of timely and affordable transport (Theme 4.3.1)<br>• Competing priorities and lack of time (Theme 4.3.2, Theme 4.5.4)<br>• Cost of accessing programs (Theme 4.5.3)<br>• Perceived unimportance of learning water safety skills for those who do not access water (Theme 4.5.1)	• Perceived importance of learning water safety skills (Theme 4.2.1, Theme 4.4.3)<br>• Availability of water safety signs to improve awareness of water safety risks (Theme 4.2.2)<br>• Perceived safety in enclosed water bodies such as pools (Theme 4.2.3)<br>• Presence of lifeguards at beaches and in pools (Theme 4.2.3)<br>• Social networks and community support from fellow migrants (Theme 4.4.1, Theme 4.5.1))<br>• Perception of poor swimming capability and previous drowning experiences (Theme 4.4.2)<br>• Use of regional languages in training (Theme 4.4.4)<br>• Communication of available programs through university and community channels (Theme 4.4.5)

	• Culturally sensitive programs (Theme 4.5.5)

• Include only the quotes/representative passages from the informants (for the specific identified theme) and exclude the question asked by the interviewer

These have been removed.

5. Discussion

• Lines 14-16: Cite published article/s about multisectoral partnerships applicable in this study

Two additional citations have been added, and the paragraph updated as:

Multisectoral partnerships are also crucial to address drowning risks [13], where the Immigration Department may also consider sharing water safety and program information with incoming migrants before they arrive in Australia, and local municipalities and Department of Education may support implementation of programs through community pools, schools and universities [14].

[13] Scarr J, Jagnoor J. Identifying opportunities for multisectoral action for drowning prevention: a scoping review, Inj Prev 2022;28:585-594.
[14] Willcox-Pidgeon S, Franklin RC, Devine S. Drowning prevention strategies for migrant adults in Australia: a qualitative multiple case study. BMC Public Health 2025;25:1911. https://doi.org/10.1186/s12889-025-23104-5
Aidoo-Frimpong G, Turner D, Collins RL

• Lines 37-45: Cite literature supporting the statements/discussion written in this paragraph

The paragraph has been updated as follows:

The content of communication should be tailored to the priorities of the target groups [17]. In our study, we found that focussing on the relevance of water safety knowledge to fishing may encourage participation of Korean and Chinese migrants. When targeting men, messaging may focus on the sport and fitness aspect of swimming. Promotions aiming to raise program awareness among migrants could emphasise the importance of water safety skills as pathway to enjoying Australian culture and lifestyle, contributing to social cohesion, employment outcomes, and youth engagement, a suggestion that has been made in other studies of migrant perceptions [18]. To encourage those who have a fear of drowning, messaging could also focus on the methods used by practitioners to ensure a safe swim environment and cultural sensitivity during classes [19, 20].

[18] Willcox-Pidgeon S, Devine SG, Franklin RC. Adult migrants urgent need for drowning prevention in Australia: water safety perceptions, attitudes, and behaviours. Health Promot Int 2025;40:4. https://doi.org/10.1093/heapro/daaf109
[19] Luckhaus JL. “Learning to swim is like learning Swedish”: A Grounded Theory study of immigrant swim teacher’s conceptualization of swimming ability and their strategies used for newly immigrated families. Soc Med Glob Health 2019; 8990244.
[20] Savage MA, franklin RC. Exploring the Delivery of Swimming and Water Safety Teacher Training to Culturally and Linguistically Diverse Communities. Int Aquat Res 2016; 9:3. https://doi.org/10.25035/ijare.09.03.03.

VERSION 2 - REVIEW

Reviewer 2

Name Guevarra, Jonathan

Affiliation University of the Philippines Manila, Department of Health Promotion and Education, College of Public Health, University of the Philippines Manila

Date 09-Oct-2025

COI

3.2 Study Design

The study applied a phenomenological approach to capture informant lived experienced.........informants' lived experiences.

Table 2 summarises the key enablers and barriers identified in the analysis and the themes where these are discussion in detail below. ....where these are discussed in detail.

Line 46-53: Please review if this should fall under 4.1.1: Exposure to water.

Please review the translated representative quotes for completeness of the statement, errors in grammar. Please remove the term "Informant" at the beginning of the quotes (for some representative quotes).

VERSION 2 - AUTHOR RESPONSE

Reviewer 2

3.2 Study Design
The study applied a phenomenological approach to capture informant lived experienced.........informants' lived experiences. Table 2 summarises the key enablers and barriers identified in the analysis and the themes where these are discussion in detail below. ....where these are discussed in detail.

The themes are discussed in detail in Section 4.11 onwards. Table 2 provides a summary of the themes by their title, and the following results section discusses these in details.

To make this clearer, we have changed the sentence and Table heading.

"Table 2 lists the key enablers and barriers identified in the analysis. These are discussed in detail in the results section below.

Table 2: Key barriers and enablers to program participation"

Line 46-53: Please review if this should fall under 4.1.1: Exposure to water.

Thank you for the suggestion. We have shifted the discussion around CPR training and exposure under a new theme 4.4.6 - Occupational requirements. This section better describes occupational training as an enabler of engagement in CPR training.

"4.4.6 Occupational requirements

Formal work or course requirements acted as an enabler to learning water safety practices. Cardiopulmonary resuscitation (CPR) training was only taken by those who required the qualifications for university courses or employment. These trainings were not drowning or water-safety specific but incorporated some elements that could be transferred to water safety.

"[Drowning rescue] training? No, not really, because I am studying. But recently I have studied individual sportin which I did a certificate in aged care. In that I learnt CPR and other skills because it has [relevance to] jobs in the medical field later." – ID4 (Male, Indian, 20s)"

Please review the translated representative quotes for completeness of the statement, errors in grammar. Please remove the term "Informant" at the beginning of the quotes (for some representative quotes).

All quotations have been edited for grammar and clarity.
